# Supplementary material for: The first wave of COVID-19 in Israel—Initial analysis of publicly available data
Source: PLoS One. 2020 Oct 29;15(10):e0240393. doi: 10.1371/journal.pone.0240393 (PMC7595440; doi:10.1371/journal.pone.0240393)
Supplement: Attachment — Submitted filename: Response to Reviewers.docx [file pone.0240393.s001.docx]

Response to Reviewers

PONE-D-20-13386

**I am extremely grateful to the anonymous reviewers for their insightful comments and suggestions.**

Reviewer #1: While I found the article interesting and well developed, I personally believe it is too descriptive at this stage and very limited in the scope of analysis. While comparing Israel and Sweden because of similar size might sound compelling at first, more rigorous tests and comparisons are needed. The two countries have very different characteristics in terms of climate, culture, population density, rural vs. urban living, etc. - Hence, if a resubmission is intended, additional countries should be added.

**Response**: Totally agree. The analysis now includes four countries: Israel, Greece, Italy, and Sweden.

Reviewer #2: This article studies the data available regarding COVID-19 infections and its fatality rate for Israel and Sweden. The aim of the study is to understand the effect of the implemented interventions and to foresee possible future scenarios.
It is an interesting study, but I have some comments on the descriptive analysis implemented.

1. The authors argue that they compare Israel and Sweden because they are countries of similar size. However, these two countries differ in many things that can affect on the evolution of the COVID19 contagion. As the author recognise, social distance is significantly different in both countries. However, there are many other substantial differences that can affect on the COVID19 dynamics such as the weather, the population density, the ability to telework, the general trust and the trust in government. It is important to discuss these substantial differences and its effect on the COVID19 dynamics.

**Response**: The differences between the four analyzed countries are now discussed in the Introduction and the Results sections.

2. The analysis would be much richer if they use data at some subnational level. It would be nice to see the analysis at the regional level. This will allow the authors also to control for some of the previous mentioned characteristics that can affect the COVI19 dynamics and that are different over the different regions in the country.

**Response**: An excellent idea! The paper now includes regional level analysis for Israel (see Fig. 2) and Italy (see Fig. 5).

3. The linear regression that they do to estimate the correlation between confirmed cases and death should be improved. There are many characteristics that can affect in the relation of these two variables, such as the capacity of the health sector and the aging of the population. It would be very interesting if the authors implement the analysis at a regional level and departing from their initial regression, they include in a parsimonious way other control variables that can effect on the relation of these two variables. This would provide a better capacity to foresee the future scenarios depending on the regional characteristics.

**Response**: The correlation between confirmed cases and deaths is now explored with respect to the time dimension. Unfortunately, the effect of additional important characteristics suggested by the reviewer could not be analyzed due to the unavailability of sufficiently detailed data on the countries included in this study.

4. In equation [1], the authors should replace R that refers to recovered people by another letter. It is misleading with R0

**Response**: In Eq. 1, R was replaced with H, for Healed, to avoid confusion with R0.
