# Supplementary material for: The first wave of COVID-19 in Israel—Initial analysis of publicly available data
Source: PLoS One. 2020 Oct 29;15(10):e0240393. doi: 10.1371/journal.pone.0240393 (PMC7595440; doi:10.1371/journal.pone.0240393)
Supplement: Attachment — Submitted filename: Response to Reviewers2.docx [file pone.0240393.s002.docx]

Response to Reviewers

PONE-D-20-13386R1

**I am again extremely grateful to the anonymous reviewers for their additional comments and suggestions.**

**Reviewer #3**: I read it with interest and enjoyed the clarity and brevity of your analysis. Nonetheless, I have decided not to accept immediately. While I found your analysis and literature to be interesting, the study would resonate more among our readers if it offered insights into the current literature by acknowledging these followings papers, which were focused on the Vietnam, lauded as the coronavirus fighting in the first wave. I believe that these measures as well as the study would have merits for the authors to enhance their current literature:

[a] Huynh, T. L. D. (2020). Does culture matter social distancing under the COVID-19 pandemic?. Safety Science, 104872.

[b] Huynh, T. L. D. (2020). “If You Wear a Mask, Then You Must Know How to Use It and Dispose of It Properly!”: A Survey Study in Vietnam. Review of Behavioral Economics, 7(2), 145-158.

[c] Huynh, T. L. D. (2020). The COVID-19 containment in Vietnam: What are we doing?. Journal of Global Health, 10(1).

[d] Huynh, T. L. (2020). The COVID-19 risk perception: A survey on socioeconomics and media attention. Econ. Bull, 40(1), 758-764.

[e] Huynh, T. L. D. (2020). Data for understanding the risk perception of COVID-19 from Vietnamese sample. Data in Brief, 105530.

After that, this manuscript is ready to be published.

**Response**: I found the above references very useful and extended the literature survey in the Introduction section accordingly.

**Reviewer #4**: Related to the 4th point by Reviewer 2, there are still a couple of places "R0" was written, for example p2, p12, p13. Please check and correct.

**Response**: checked and corrected as necessary.

**Reviewer #5**: This article explores the evolution of Covid-19 in Israel compared to Greece, Italy and Sweden, and aims to understand the effect of the implemented interventions and to predict possible future scenarios. Even though it is mostly at descriptive level, concerning that every single piece of analysis adds to the information set of humanity, it might be valuable to publish it, once the necessary implementations are made.
It seems that the author successfully implemented most of the recommendations of the referees. He added two more countries, Greece and Italy, and two subnational analysis, one for Israel and one for Italy. Additionally, the graphs and the regression analyses are clearer and that`s why make more sense compared to the initial submission. Thus, I think the paper has improved a lot.
I would recommend couple of minor additions:
1. The “Current mortality rate” needs an explanation of what that current date is. Plus a “current number of confirmed cases per 100K” would be added to Table 1 to give a clearer picture.

**Response:** The word “current” was replaced with specific dates, where applicable (including Table 1). The number of confirmed cases was added to Table 1 as well.

2. The y-axis of Figure 2 is not stated. The author should add a title to that axis.

**Response**: The y-axis title added.

3. I do not think the author needs to spend 3-4 sentences per figure to explain everything in the Figures 3-4-5. They are already very clear. He can safely remove some. This will shorten the paper a little.

**Response**: Several sentences removed.

Once these minor additions are added, I think the paper will add to the literature and can be published
